# Obstructive Sleep Apnea and Hypertension: A Review of the Relationship and Pathogenic Association

**DOI:** 10.7759/cureus.8241

**Published:** 2020-05-22

**Authors:** Amna Bangash, Fareha Wajid, Raju Poolacherla, Fatiha Kabir Mim, Ian H Rutkofsky

**Affiliations:** 1 Internal Medicine, California Institute of Behavioral Neurosciences and Psychology, Fairfield, USA; 2 Anesthesia and Perioperative Medicine, California Institute of Behavioral Neurosciences and Psychology, Fairfield, USA; 3 Pediatric Anesthesia and Pain, Western University, London, CAN; 4 General Practice, California Institute of Behavioral Neurosciences and Psychology, Fairfield, USA; 5 Psychiatry, California Institute of Behavioral Neurosciences and Psychology, Fairfield, USA

**Keywords:** sleep apnea, essential hypertension, obesity, snoring, continuous positive airway pressure (cpap), sleep apnea and hypertension

## Abstract

Obstructive sleep apnea (OSA) is defined as episodes of hypopnea or apnea, which leads to a partial or complete block of airways. Hypertension, on the other hand, is defined as an increase in systemic arterial blood pressure to a certain threshold. OSA and hypertension share many common factors in pathophysiology, such as gender, obesity, unhealthy lifestyle, impaired quality of sleep, renin-angiotensin system, and increased fluid distribution. In order to manage our patients effectively, we need to explore further the correlation between the two conditions.

## Introduction and background

Obstructive sleep apnea (OSA) is defined as episodes of obstruction of the upper airway during sleep, with a minimum prerequisite frequency of five events per hour and lasting for at least 10 seconds. It can lead to decreased breathing or even complete cessation of breathing causing temporarily low oxygen and high carbon dioxide in the body [1,2]. There are several types of sleep apnea, but the most common is OSA. This type of apnea occurs when throat muscles intermittently relax and block the airway during sleep. A noticeable sign of OSA is snoring. Sleep apnea has a strong association with cardiovascular diseases, diabetes, and stroke [3]. It is increasing in developed countries with an increase in the obesity rate. It is known to affect 24%-26% of men and 17%-28% of women between 30 and 70 years of age [4].

Hypertension (HTN) is defined as the elevation of arterial blood pressure (BP) above a certain value, systolic of equal to or greater than 140 mmHg and diastolic of equal to or greater than 90 mmHg [1]. BP is the force that the blood exerts against the walls of the blood vessels. It can be classified as primary or secondary HTN depending upon the cause. Obesity, older age, high salt diet, diabetes, and sedentary lifestyle kidney diseases are the risk factors. There are various stages of HTN. The most initial step in the treatment of HTN is lifestyle modification followed by medications of different classes that help in lowering BP. OSA is more common in patients with HTN than in the general population, and many patients with OSA may have HTN as well [5]. OSA and HTN are interrelated diseases, and approximately 75% of treatment-resistant hypertension (TRH) patients have an underlying OSA [6].

An increase in the development of HTN in the general population requires an in-depth analysis of the association of HTN with other possible co-existing conditions, such as sleep apnea, obesity, and hypercholesterolemia. A detailed analysis of the association can help us improve the investigations and treatment protocols of the patients. It can further help us to prevent the development of these closely interrelated conditions.

Method

PubMed was systemically searched for related articles about OSA and its association with HTN. Keywords used were sleep apnea, essential hypertension, obesity, snoring, continuous positive airway pressure (CPAP), and sleep apnea and hypertension. In our research, we excluded articles that are 10 years or older, 9677 articles were found for sleep apnea and 3457 articles were found for essential hypertension. Upon searching PubMed for the keyword obesity, 88,431 articles were found. The search for keyword snoring yielded 1242 articles, whereas CPAP yielded 2811 articles. The keyword sleep apnea and hypertension yielded 1456 articles.

## Review

Association between HTN and sleep apnea

**Figure 1:**
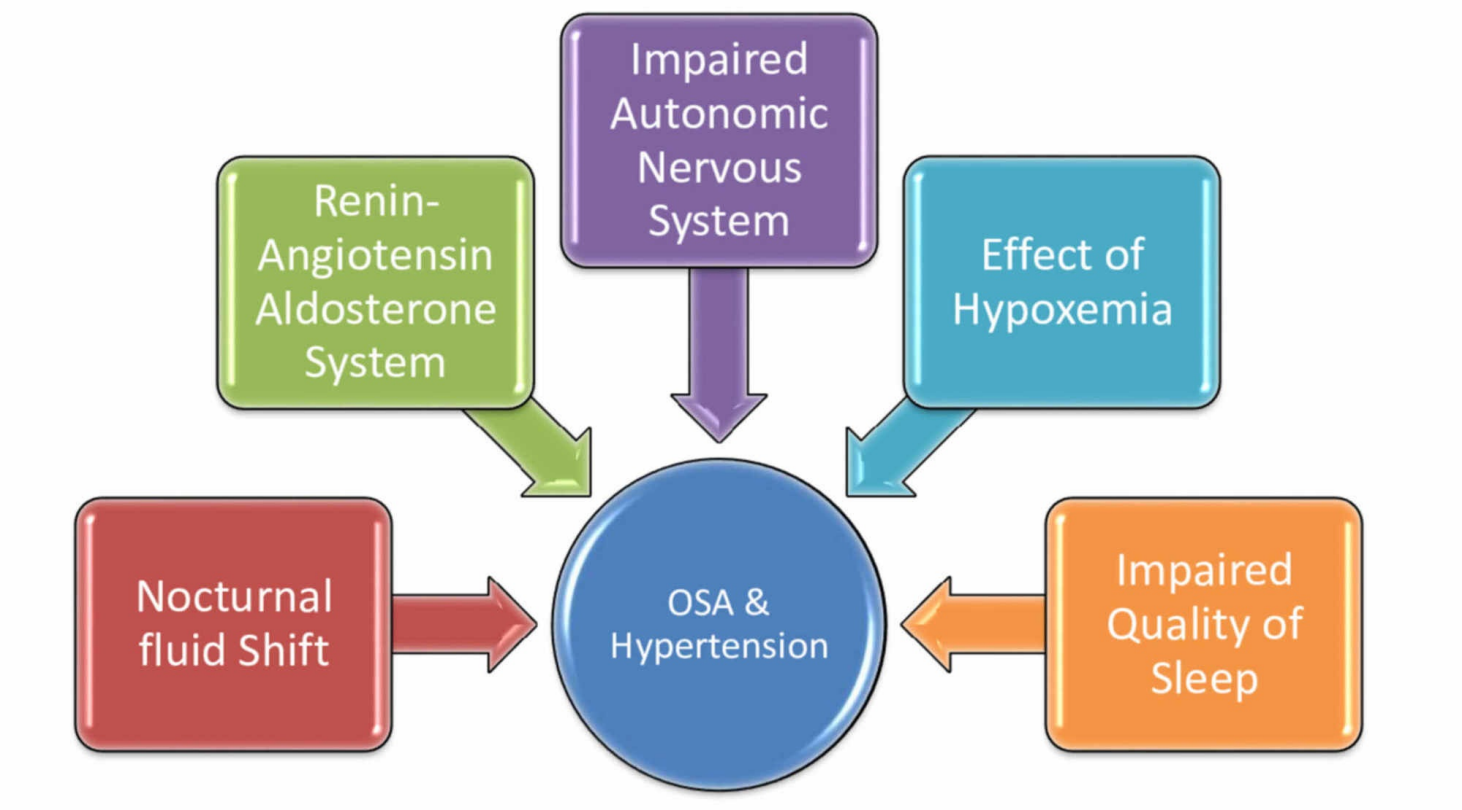
Factors relating hypertension and obstructive sleep apnea (OSA)

The pathophysiology of HTN in OSA is complex and is dependent on various factors, such as an increase in sympathetic tone, peripheral vasoconstriction, increased renin-angiotensin-aldosterone activity, and altered baroreceptor reflexes [4]. The factors linking the pathophysiology of HTN and OSA are hypoxemia, nocturnal fluid shift, an increase in sympathetic tone with a decrease in parasympathetic tone, impaired quality of sleep, and renin-angiotensin-aldosterone system (Figure 1).

Nocturnal fluid shift

At night, the fluid from the lower limbs is redistributed to the neck, which causes further obstruction and rise in BP and episodes of hypopnea or hypoxia in patients with HTN and OSA. Furthermore, the increased levels of aldosterone enhance fluid retention, thus increasing the upper airway obstruction [7].

Renin-angiotensin-aldosterone system

Renin-angiotensin-aldosterone is a hormonal system that regulates BP, fluid, and electrolyte balance in the body. Renin produced from the kidneys, along with enzymes, causes the conversion of angiotensinogen to angiotensin I, which is further converted to angiotensin II. Angiotensin II is a strong vasoconstrictive peptide that causes vasoconstriction and increases the BP. The recurrent episodes of obstruction of the upper airway lead to hypoxia, which in turn leads to increased activation of renin. A 2016 meta-analysis of 13 studies revealed that patients with OSA have higher levels of angiotensin II and aldosterone, especially in cases with co-existing HTN [8]. Excess levels of aldosterone cause edema of nasopharyngeal tissues and upper airways, which in turn leads to airway obstruction and further progression of OSA [9]. CPAP therapy is found to be associated with downregulation of renal renin-angiotensin system activity [10].

Impaired sleep quality

OSA is one of the most important causes of impaired sleep quality [11]. Impaired sleep or deficiency of sleep is also a potential contributor to HTN. A longitudinal study was conducted, and it was concluded that chronic insomniacs with short sleep duration were at increased risk of incident HTN, but this effect was largely explained by controlling obesity, whereas many other studies have shown positive correlations between sleep deprivation and various adverse cardiovascular risk factors: arterial stiffness, endothelial dysfunction, sympathetic activity, non-dipping nocturnal BP pattern, and insulin insensitivity [12-16].

Role of autonomic nervous system

The sympathetic nervous system is activated and parasympathetic nervous system is deactivated by changes in the levels of CO_2_ and O_2_; the increase in CO_2_ and decrease in O_2_ are caused by the events of apnea [17]. Increased catecholamine levels along with the changes in the autonomic nervous system continue during daytime and can cause the development of HTN [18]. During postapneic hyperventilation, the increase in BP can go as high as 240/30 mmHg [19,20]. Studies on animals demonstrated that the rise in BP, which is associated with OSA events, can be undermined by renal denervation [21]. 

Cytokine-mediated effect of hypoxemia

It has been observed that OSA correlates with an increased burden of systemic inflammation and higher concentrations of high-sensitive C-reactive protein (hs-CRP), interleukin (IL)-1, IL-8, IL-6, tumor necrosis factor-alpha (TNF-α), Rantes, and sICAM [22]. The oxidative stress caused as a result of OSA acts like an ischemic reperfusion injury, leading to the release of reactive oxygen species [23,24]. This overall increase in oxidative stress leads to an increase in cardiovascular risk. It has been suggested by studies in a murine model that atorvastatin, which is known to reduce inflammation, prevented various adverse cardiovascular processes related to intermittent hypoxia [25].

Effect of age on pathophysiology of OSA

A number of studies have been reported that the effects of OSA on cardiovascular conditions such as atrial fibrillation and HTN are more prominent in younger than in older older individuals. This hypothesis that OSA affects younger versus older patients differently was assessed in a study of changes in the renal vascular resistance index but no strong differences between the age categories were found [26].

Treatment

The initial steps in the management are taken to define the baseline of the patient profile, and to check for the end-organ damage for which the patient will undergo physical examination BP monitoring [27].

Lifestyle modification

Lifestyle modification is the first and initial treatment. Obesity has an independent link with HTN and OSA, so the patients can be encouraged to adopt lifestyle modifications like weight loss. In a randomized control trial, to assess the effect of CPAP and weight reduction on OSA patients were divided into groups having CPAP treatment alone, weight reduction or CPAP, and weight reduction together [28]. A reduction in CRP, insulin, and triglyceride levels was seen in the group with both interventions; however, no such reduction in CRP level was noted in patients taking CPAP alone. Reductions in insulin resistance and triglyceride levels were greater in the combined intervention groups than in the group receiving CPAP only.

Continuous positive airway pressure

The main treatment therapy for OSA is CPAP. The adherence is still suboptimal although numerous developments in machine dynamics have been made, which includes quieter pumps, softer masks, and improved portability. Adherence to CPAP therapy remains the main problem as it low (30%-60%). Since the nature of HTN is multifactorial, the effect of CPAP is yet to be established. In patients with minimal symptoms, CPAP has a neutral effect on BP but those with resistant HTN, CPAP decreases the systolic BP by 5‐7 mmHg [29].

Upper airway surgery and oral appliances

Upper airway surgeries, such as tonsillectomy and uvulopalatopharyngoplasty (UPPP), are also being considered as treatment options for OSA surgery [4]. In mild to moderate OSA, oral appliances can be recommended as an alternative treatment to CPAP. A meta-analysis of seven studies (399 OSA patients involved) found that treatment with oral appliances was more beneficial for BP reduction than CPAP therapy. The average drop in the systolic BP and diastolic BP was 2.7 and 2.7 mmHg, respectively [30].

Future questions

Further research and work are required for an in-depth analysis of the association between HTN and sleep apnea. If HTN and sleep apnea are associated, then do all the patients with sleep apnea develop HTN? How much susceptible is every hypertensive patient to developing sleep apnea? What preventive measures can be taken at the time of diagnosis of a hypertensive patient to avoid developing sleep apnea? How frequently should a patient with sleep apnea be monitored for HTN? If sleep apnea and HTN are associated, then why not all the patients with HTN have sleep apnea? All these questions warrant further detailed investigation and research into the association of the two conditions by future scientists.

Limitations

As a traditional review article summarizes the already published studies, not all the possible new facts and analysis were included. The search was limited to studies that 10 years older, primarily focusing on the study of the association between the two conditions.

## Conclusions

The apneic episodes in OSA lead to an increase in BP, which further exposes the patient to cardiovascular and cerebrovascular risks. Thus, identification of the co-existent conditions is important to provide timely and proper treatment to patients. Due to the increasing number of patients with OSA, more research and studies are required to explore the co-relation of OSA with HTN as well as other conditions. In this review article, we have tried to discuss a few of the well-related and studied factors that co-relate OSA and HTN. Factors such as obesity and hyperlipidemia require further research and investigation as these factors can also be contributing to either of the two conditions .
